# Trades Unionism in High Places

**Published:** 1902-06-14

**Authors:** 


### Trades Unionism in High Places.

We think the time has come when an earnest protest should be made against the manner in which the General Medical Council deal with certain misdemeanours for the punishment of which they hesitate to apply the only punitive measure which they possess, namely, expulsion from the Register.

A practice has grown up of first of all considering the facts brought in support of the charge, and, if it is held that these are proved, adjourning the case for further consideration, in the hope, and, indeed, in the well-founded expectation that the sinner will repent before the day of final sentence comes round. He knows, or at least he thoroughly believes, that the Council possess full power as to what they may choose to call "infamous conduct," he feels that he is at their mercy, so he reforms his ways. The Council then decide to "proceed no further," and there is an end of the matter. This is an ingenious way of getting out of the difficulty, presented by the fact that the Council possess only one form of punishment for evildoers of all degrees, and when it is applied to cases over which the Council possess undoubted jurisdiction, it is both just and effective. For example, the Council are well within their powers in treating as "infamous conduct" the offences of "covering," and of employing unqualified assistants to perform work demanding professional skill. Both of these offences cut at the root of the very object for which the Council was appointed, that namely of enabling the public to distinguish between qualified and unqualified practitioners, and in regard to both the Council have felt so sure of their powers that they have given definite notice to the profession that all offenders in these respects will be expelled from the Register. But of late the term "infamous conduct" has been freely applied to an offence which has nothing to do with the original object of the Act. Under the influence of what has by some been called trades-union sentiment many efforts have been made to induce the Council to declare association with advertising, or "touting," or canvassing institutions, to be "infamous conduct," rendering the offender liable to be removed from the Register. The Council, however, refused to do anything of the sort. Time after time the matter was discussed, but the Council were sufficiently well advised to see that this offence against professional proprieties was one which could not by any legitimate stretch of language be termed "infamous." But in a weak moment, in June 1899, they were persuaded to pass the following resolution: "That the Council strongly disapprove of medical practitioners associating themselves with medical aid associations by which systematic canvassing and advertising for the purpose of procuring patients are practised." By some this pronouncement was held to be merely a pious opinion, but whether it was to be so regarded or not it was made clear by the prolonged discussion and by the care which was taken in framing the exact wording of the resolution, that the offence with which it dealt was regarded in a very different light from those of "covering," etc., as to the penalties attending which the Council had two years previously given such definite notice to the profession. Nevertheless, this resolution has of late been used as if it stood on all fours with the "notice" above referred to. Practitioners have been brought before the Council charged with "infamous conduct" in that they have accepted appointments under societies or institutions which canvass or advertise for the purpose of procuring patients; and the whole formula adopted in cases of "infamous conduct" has been gone through, including the warning and the adjournment, the resignation of his post by the offender, and the final decision "to proceed no further." We have no sympathy with advertising, or with medical aid associations, or with the people who carry on this sort of work, but we would point out that the action which the Council have lately taken in regard to them (even if strictly legal which is perhaps uncertain) amounts to sheer unmitigated bullying. If the Council are prepared to maintain that connection with these associations is "infamous" they ought in all honesty to give notice of their intention to proceed against offenders in the same terms as they have given notice in regard to matters as to which their rights are undoubted. If on the other hand they admit, as would seem to be the case from the terms of their resolution, that there is a wide difference between these offences, and if they are not prepared to proceed to extremities or to turn men off the Register for advertising, then without a shadow of doubt the hurling of all this stage thunder against these misguided practitioners is merely a bit of unscrupulous bullying and undignified bluff. When the Council come cap in hand to Parliament, as they will be forced to do before long, asking for financial assistance we shall be much surprised if someone does not let them see that they cannot safely prostitute for trades union purposes powers given them for the protection of the public.

